# Evidence for higher sensitivity of recombinant Tri a 36 compared to omega-5-gliadin for diagnosis of wheat food allergy

**DOI:** 10.1186/2045-7022-3-S3-P171

**Published:** 2013-07-25

**Authors:** A Baar, S Pahr, C Constantin, S Giavi, M Alkistib, NG Papadopoulos, C Ebner, A Mari, S Vrtala, R Valenta

**Affiliations:** 1 Department of Pathophysiology and Allergy Research, Christian Doppler Laboratory for the Development of Allergen Chips, Medical University of Vienna, Austria; 2 Department of Pathophysiology and Allergy Research, Medical University Vienna, Vienna, Austria; 3 Allergy and Immunology Research Centre, University of Athens, Athens, Greece; 4 Ambulatory for Allergy and Clinical Immunology, Ambulatory for Allergy and Clinical Immunology, Vienna, Austria; 5 Center for Molecular Allergology, IDI-IRCCS, Rome, Italy; 6 Department of Pathophysiology and Allergy Research, Christian Doppler Laboratory for Allergy Research, Medical University Vienna, Vienna, Austria

## Background

Wheat is one of the most important food allergen sources. Using natural wheat allergen extracts for serological diagnosis of wheat-induced food allergy false positive test results are frequently obtained, in particular in grass pollen allergic patients. Therefore, Tri a 19, an omega-5-gliadin, which is known as a major allergen in wheat dependent exercise induced anaphylaxis (WDEIA) and in wheat food allergy in children, is widely used for the serological diagnosis of wheat-induced food allergy.

## Methods

We have recently characterized Tri a 36, a low molecular weight glutenin as new major wheat food allergen. Here we compared recombinant Tri a 36 with ImmunoCAPs containing omega-5-gliadin for the detection of specific IgE antibodies in a population of wheat food allergic children (n=23) and grass pollen allergic patients (n=21). Wheat food allergic children had a clear clinical history of wheat food allergy with symptoms clearly attributable to wheat ingestion. Grass pollen allergic patients suffered from grass pollen-induced respiratory allergy but regularly ate wheat products without any clinical symptoms. Sera from both populations were tested by ImmunoCAPs containing wheat extract or omega-5-gliadin and by IgE ELISA to rTri a 36.

## Results

Using wheat extract-based ImmunoCAPs all but one of the wheat food allergic patients showed allergen-specific IgE levels >0.35 kUA/L but 17 out of the 21 grass pollen allergic patients gave false positive test results. Eleven (i.e.,48%) of the wheat food allergic patients showed >0.35 kUA/L IgE against omega-5-gliadin and two of the grass pollen allergic patients gave false positive test results. Using rTri a 36, 14 (i.e., 61%) of the wheat food allergic patients were diagnosed and only one grass pollen allergic patient gave a false positive test result. Each of the sera from the wheat food allergic patients with IgE reactivity to omega-5-gliadin also reacted with rTri a 36.

## Conclusion

Our results indicate that rTri a 36 has higher sensitivity and specificity than omega-5-gliadin for the diagnosis of wheat food allergy.

## Disclosure of interest

None declared.

